# Improving stool sample processing and pyrosequencing for quantifying benzimidazole resistance alleles in *Trichuris trichiura* and *Necator americanus* pooled eggs

**DOI:** 10.1186/s13071-021-04941-w

**Published:** 2021-09-25

**Authors:** Javier Gandasegui, Berta Grau-Pujol, María Cambra-Pelleja, Valdemiro Escola, Maria Antonietta Demontis, Anelsio Cossa, José Carlos Jamine, Rafael Balaña-Fouce, Lisette van Lieshout, José Muñoz, María Martínez-Valladares, The Stopping Transmission of Intestinal Parasites (STOP) Project Consortium

**Affiliations:** 1 Instituto de Ganadería de Montaña (CSIC-Universidad de León), Grulleros, León Spain; 2 Departamento de Sanidad Animal, Facultad de Veterinaria, Universidad de León, Campus de Vegazana, León, Spain; 3 ISGlobal, Hospital Clínic - Universitat de Barcelona, Barcelona, Spain; 4 Centro de Investigação Em Saúde de Manhiça (CISM), Maputo, Mozambique; 5 Fundación Mundo Sano, Buenos Aires, Argentina; 6 Departement of Parasitology, Leiden University Medical Center, Leiden, The Netherlands; 7 Departamento de Ciencias Biomédicas, Universidad de León, León, Spain

**Keywords:** Soil-transmitted helminths, Benzimidazoles, Anthelmintic resistance, Pyrosequencing

## Abstract

**Background:**

There is an urgent need for an extensive evaluation of benzimidazole efficacy in humans. In veterinary science, benzimidazole resistance has been mainly associated with three single-nucleotide polymorphisms (SNPs) in the isotype-1 β-tubulin gene. In this study, we optimized the stool sample processing methodology and resistance allele frequency assessment in *Trichuris trichiura* and *Necator americanus* anthelmintic-related SNPs by pyrosequencing, and standardized it for large-scale benzimidazole efficacy screening use.

**Methods:**

Three different protocols for stool sample processing were compared in 19 *T. trichiura*-positive samples: fresh stool, egg concentration using metallic sieves with decreasing pore size, and egg concentration followed by flotation with saturated salt solution. Yield of each protocol was assessed by estimating the load of parasite DNA by real-time PCR. Then, we sequenced a DNA fragment of the β-tubulin gene containing the putative benzimidazole resistance SNPs in *T. trichiura* and *N. americanus.* Afterwards, resistant and susceptible-type plasmids were produced and mixed at different proportions, simulating different resistance levels. These mixtures were used to compare previously described pyrosequencing assays with processes newly designed by our own group. Once the stool sample processing and the pyrosequencing methodology was defined, the utility of the protocols was assessed by measuring the frequencies of putative resistance SNPs in 15 *T. trichiura*- and 15 *N. americanus*-positive stool samples.

**Results:**

The highest DNA load was provided by egg concentration using metallic sieves with decreasing pore size. Sequencing information of the β-tubulin gene in Mozambican specimens was highly similar to the sequences previously reported, for *T. trichiura* and *N. americanus*, despite the origin of the sample. When we compared pyrosequencing assays using plasmids constructs, primers designed in this study provided the most accurate SNP frequencies. When pooled egg samples were analysed, none of resistant SNPs were observed in *T. trichiura*, whereas 17% of the resistant SNPs at codon 198 were found in one *N. americanus* sample.

**Conclusions:**

We optimized the sample processing methodology and standardized pyrosequencing in soil-transmitted helminth (STH) pooled eggs. These protocols could be used in STH large-scale screenings or anthelmintic efficacy trials.

**Graphical Abstract:**

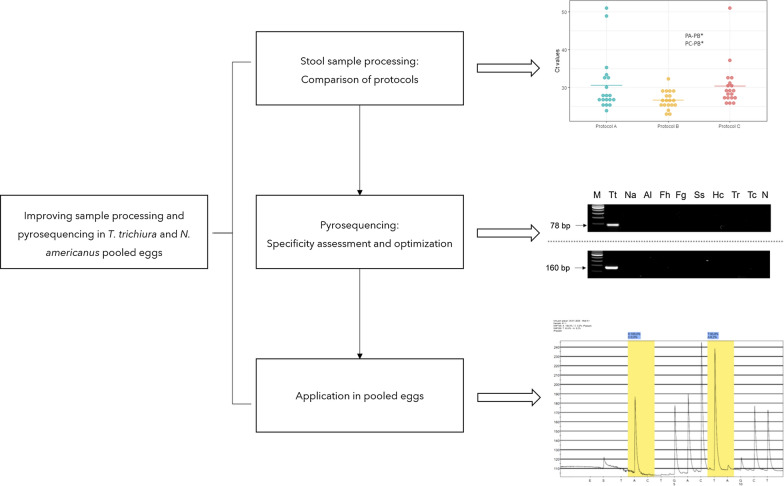

**Supplementary Information:**

The online version contains supplementary material available at 10.1186/s13071-021-04941-w.

## Background

Neglected tropical diseases affect populations in the world’s poorest communities. Among them, soil-transmitted helminth (STH) infections (ascariasis, trichuriasis, and hookworm disease) are the most prevalent with approximately 1.5 billion people infected worldwide [1]. The current World Health Organization (WHO) control strategy for STH emphasizes morbidity control through mass drug administration (MDA) targeting pre-school and school-aged children, women of childbearing age, and adults in certain high-risk occupations [2]. The drugs of choice for MDA campaigns are benzimidazoles, mainly albendazole and mebendazole, which are administered once or twice a year depending on the prevalence of the disease [2]. As observed in veterinary practice, the widespread use of anthelmintic drugs can entail a great selection pressure for resistant parasite strains, thus decreasing efficacy and worsening treatment response [3]. Considering that the efficacy of benzimidazole has been significantly reduced for *Trichuris trichiura* and hookworm over the past few decades [4, 5], the possible emergence of anthelmintic resistance is now a major concern in the treatment and control of STH infections [6].

Benzimidazole resistance in veterinary gastrointestinal nematodes, mostly those infecting ruminants, has been mainly associated with three single-nucleotide polymorphisms (SNPs) in the isotype-1 β-tubulin gene, causing an amino acid substitution of phenylalanine to tyrosine at codons 200 (F200Y) and 167 (F167Y), and a glutamate-to-alanine change at codon 198 (E198A) [6]. In STH, the association of these genetic markers with benzimidazole resistance is still unclear [7, 8] and only poorly reported for both *T. trichiura* [9–11] and hookworm [10–15]. Most of these studies just describe the presence or absence of these SNPs and did not attempt to associate them with the response to treatment with benzimidazoles. Consequently, there is an urgent need for sensitive and accurate tools to detect the presence of these SNPs and standardize the detection of markers of benzimidazole resistance in STH.

Several DNA detection-based tools have been used for detecting benzimidazole resistance markers in STH, including RFLP-PCR [12], q-PCR [13, 14], LAMP [15], and pyrosequencing [9, 10]. The pyrosequencing method is more accurate compared with other molecular tools, mainly because it is quantitative [7]. In addition, it is also able to determine the frequencies of multiple resistance alleles in the same run when they are close in the genome, such as putative benzimidazole resistance SNPs in codons 198 and 200.

In veterinary science, pyrosequencing has been used extensively to assess the relation of putative resistance SNPs to benzimidazole efficacy. The main outcomes of these resistance studies were to evaluate whether resistance SNP frequencies could be used to predict treatment response and to evaluate the variation of these frequencies before and after treatment [16, 17]. Additionally, detection of SNPs in veterinary practice is often carried out in pooled eggs or larvae isolated from faecal samples, which provides frequencies within nematode subpopulations infecting one individual or at farm level [16, 18, 19]. Thus, sample collection and analysis are quicker and simpler compared to the analysis of single parasites, whether adults, larvae, or eggs, since the genetic markers are assessed in a large number of individuals simultaneously. This methodology allows faster genotypic studies and makes them suitable for drug efficacy trials or large-scale anthelmintic resistance surveillance.

In human STH infections, various pyrosequencing methods have been described for the detection of benzimidazole resistance-associated SNPs [9–11]. However, SNP detection was frequently performed in single eggs [9, 10], making protocols less suitable for larger studies. Thus, the use of pooled eggs instead of single individuals is desirable for large-scale monitoring, and consequently, protocols for processing samples and testing resistance markers should be evaluated and improved. Therefore, the main aim of this study was to optimize and standardize a methodology for the processing of human stool samples and subsequent assessment of resistance allele frequencies by pyrosequencing in *T. trichiura* and *Necator americanus* pooled eggs. This could contribute to and facilitate the implementation of large-scale anthelmintic resistance monitoring.

## Methods

### Sample collection and parasitological examination

Stool samples used to conduct this research were collected during the development of the MARS and WASH-IT studies, both conducted in the same research area: Manhiça district, Southern Mozambique. The aim of the MARS study was to assess responses to anthelmintic treatment and the prevalence of anthelmintic resistance to albendazole in Manhiça district. The aim of the WASH-IT study was to evaluate the association between a water, sanitation, and hygiene intervention at schools and in the community and STH infection. The sample collection methodology in the MARS and WASH-IT studies was identical. Each participant was given a parasitological flask for stool collection. Samples were collected on a second visit the following morning. A single stool sample was individually obtained from each participant. After collection, samples were transported to the Manhiça Health Research Centre (CISM) for detection of STH by duplicate Kato–Katz thick smear [20].

The MARS study was carried out from December 2017 to February 2019. After detection of STH, 2 g of the remaining fresh stool sample was subsequently kept at −80 °C until being sent to the Department of Animal Health, University of León, Spain. Once there, all samples were analysed again by triplex PCR for the simultaneous detection of *T. trichiura*, *N. americanus*, and *Ascaris lumbricoides*, following the methodology previously described [21]. Then, we retrieved a set of samples positive by Kato–Katz and/or triplex PCR tests. Thus, we selected 15 *N. americanus*- and 93 *T. trichiura*-positive samples. Of the 93 *T. trichiura*-positive samples, 78 were randomly allocated in eight different batches, and subsequently, eight pooled samples of 7 g each were prepared. Then, pooled samples were processed using the different protocols described below, in order to optimize the sample processing. Aliquots from each stool sample were stored at −80 °C until DNA extraction. Additionally, the remaining 15 *T. trichiura*- and 15 *N. americanus*-positive samples were analysed by pyrosequencing at the University of León after the optimization of stool sample processing.

**Fig. 1:**
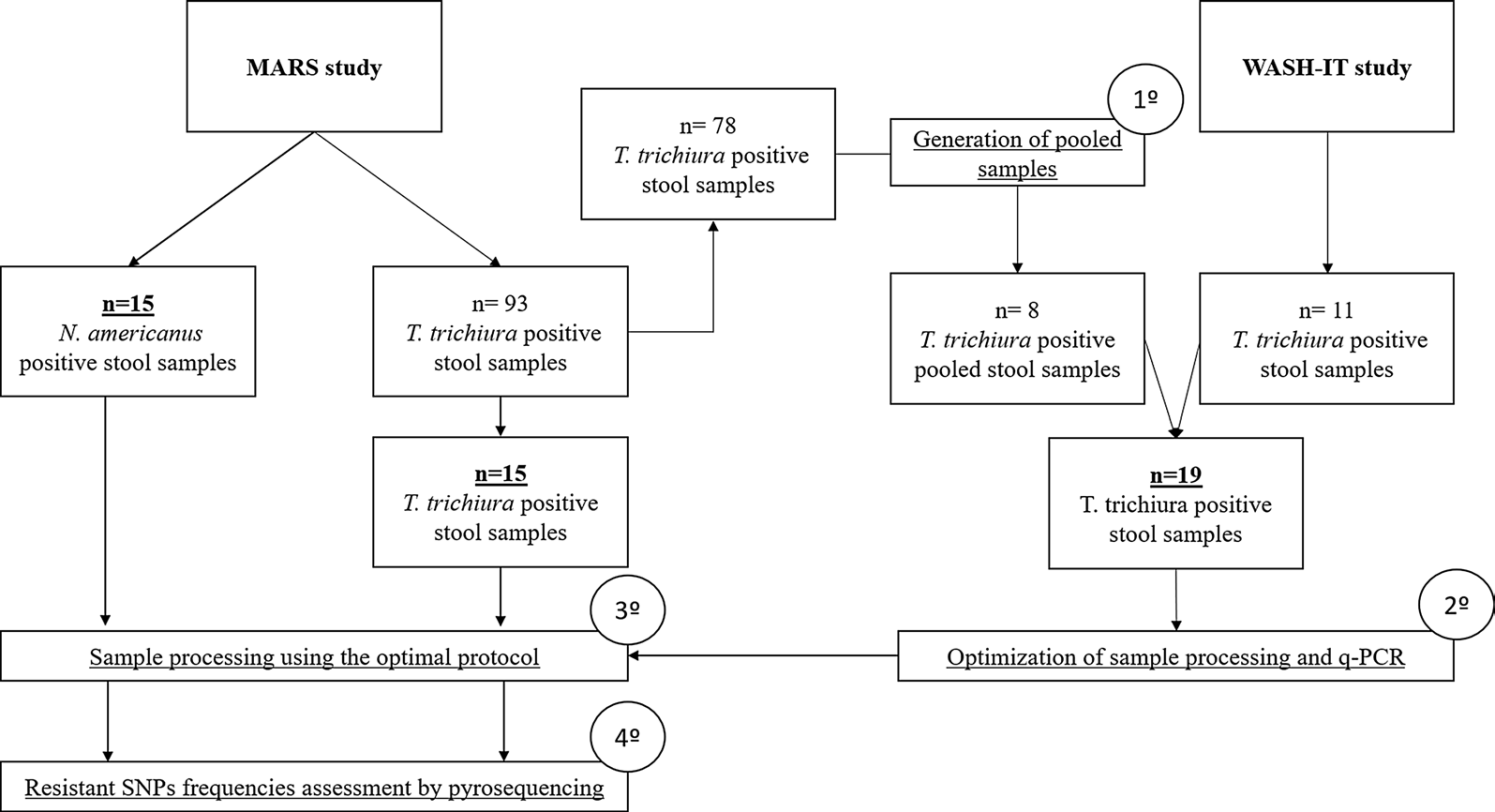
Samples flow chart. 1º to 4º indicates the sequence of the workflow

The WASH-IT study was developed from March 2019 to February 2020. After detection of STH, 11 *T. trichiura*-positive samples were immediately processed at CISM using the different protocols described below. The resulting aliquots were stored at −80 °C until being sent to the University of León for DNA extraction. Figure 1 summarizes the flow of the stool samples.

### Optimization of sample processing

#### Protocols for processing *T. trichiura*-positive stool samples

A total of 19 *T. trichiura*-positive stool samples (8 pooled and 11 non-pooled samples) were used to optimize sample processing. Three aliquots of each stool sample were prepared according to the following protocols before their storage for later DNA extraction: unprocessed stool samples, protocol A (PA); egg concentration using metallic sieves, protocol B (PB); and egg concentration and later flotation with saturated salt solution, protocol C (PC).

For PA, stool samples were exhaustively homogenized using a spatula; 250 mg of each unprocessed sample was weighed and stored at −80 °C.

In the case of PB, 3 g of fresh stool sample was weighed and homogenized after shaking with water and glass beads in a 50-ml bottle for 1–2 min. Then, the homogenized sample was forced through three metallic sieves of decreasing pore size (150, 80, and 20 µm) using high-pressure tap water. The sediment retained in the 20-µm sieve contained the *T. trichiura* eggs; 250 mg of this sediment was weighed and stored at −80 °C.

Finally, PC was the same as PB but included some additional steps; the sediment retained in the 20-µm sieve was transferred to 15-ml tubes and centrifuged at 2500 rpm. The supernatant was removed, and the sediment was mixed with a saturated solution of sodium chloride (density = 1.2 g/ml). The tube was centrifuged at 2500 rpm, and then the complete supernatant was passed through the 20-µm sieve. The sediment retained in the sieve was washed with water to remove the saturated salt solution. All sediment was stored at −80 °C for later DNA extraction.

The three protocols were applied to the 19 *T. trichiura*-positive samples, thus obtaining 57 aliquots (three per sample). A detailed description of the procedures for sample processing can be found in the Additional file 1.

#### DNA extraction

DNA was extracted using a PowerFecal DNA extraction kit (QIAGEN) following the manufacturer’s instructions. The same protocol was used for the 57 aliquots obtained from the *T. trichiura*-positive samples. DNA samples were preserved at −20 °C until use.

#### Measurement of *T. trichiura* DNA load by real-time PCR

Twenty microlitres of DNA extracted from the aliquots previously obtained was sent to Leiden University Medical Centre to be analysed by real-time PCR. The yield of each protocol was assessed by estimating the parasite DNA concentration as previously described [22]. Briefly, multiplex real-time PCR detection was used to detect and quantify parasite-specific DNA of two helminth species, *Schistosoma* sp. and *T. trichiura* [22]. The cycle threshold (Ct) values obtained reflected the parasite-specific DNA load in the sample tested. As we analysed the 57 aliquots, we estimated three different Ct values for each sample, corresponding to each protocol for sample processing.

### Determination of β-tubulin gene sequence from Mozambican specimens

One *N. americanus*-positive sample and one *T. trichiura*-positive sample collected during MARS were used to isolate parasite eggs. The isolation was done visually by picking up each egg using a 10-μl pipette from a petri dish, under a microscope. Ten individual eggs and a pool of 50 eggs of both species were retrieved and placed into a PCR tube. Then, genomic DNA from each individual and pooled eggs was extracted using a PowerFecal DNA extraction kit (QIAGEN).

A DNA fragment containing the three resistance-associated codons (167, 198, and 200) of each species was amplified by PCR and then sequenced. Primers were designed based on the sequencing information available for the β-tubulin gene in the NCBI database for *T. trichiura* (AF034219) and the isotype-1 β-tubulin gene for *N. americanus* (EF392851), and using Primer3Plus online software [23]. In order to obtain amplification and sequencing information from the larger number of eggs, two different primer pairs were designed for each DNA sequence. For *N. americanus*, we amplified two overlapping fragments due to the large size of the intron between codons 167 and 198 of the isotype-1 β-tubulin gene to simplify the Sanger sequencing. The PCR was carried out using the commercial DNA AmpliTools HotSplit Master Mix (BioTools) in a final volume of 20 µl and the primer concentration was 0.5 µM each. Primers and PCR conditions are shown in the Additional file 2: Tables S1 and S2, respectively. PCR products were monitored by 1.5% agarose gel electrophoresis. The specific amplified fragments were purified using a SpeedTools Clean-up kit (BioTools), and Sanger sequencing was done at the University of León Sequencing Facility. The sequences obtained were aligned using DNAstar software and the consensus sequence for each species was recovered.

### Design and production of susceptible-type (ST) and resistant-type (RT) plasmids

Based on the resulting sequences from Mozambican specimens, two different types of plasmid for each STH were designed, one with the susceptible genotype for the three SNPs (ST) and another with the resistant genotype (RT). The plasmids including the sequences of interest were synthesized by GeneCust (France). Plasmids were transformed into XL1-Blue *Escherichia coli* (Agilent)-competent cells and selected on Luria broth (LB) (Thermo Fisher) agar plates supplemented with 0.1 mg/ml ampicillin (Sigma Aldrich). Then, one single colony with one construct was inoculated in LB medium for large-scale culture. Plasmid DNA was extracted using a SpeedTools Plasmid DNA Purification kit (BioTools) and the DNA sequence of each construct was confirmed by Sanger sequencing at the University of León Sequencing Facility.

### Pyrosequencing assays

#### Pyrosequencing optimization with the plasmid constructs

In order to optimize the pyrosequencing assays, we attempted to estimate the SNP frequencies in the plasmid constructs. Thus, for each species, plasmid DNA from RT/ST plasmids was extracted and then mixed together at different proportions of 0%, 30%, 50%, 70%, and 100%, simulating different resistance levels (10–30% low, 30–50% medium–low, 50–70% medium–high, and 70–100% high). For this purpose, the plasmid DNA was extracted using a SpeedTools Plasmid DNA Purification kit (BioTools) and then its concentration was measured in triplicate using a NanoDrop ND-100 spectrophotometer (Thermo Fisher Scientific) to obtain an average value. Plasmid DNA mixtures were prepared and adjusted to a final concentration of 1 ng/µl; then, tenfold serial dilutions were prepared in order to work using smaller DNA amounts and thus avoid cross-contamination. These plasmid DNA mixtures were used for assessing the accuracy of the different pyrosequencing assays.

Two different sets of primers for each species were used for amplifying two small fragments of isotype-1 β-tubulin, one surrounding the SNP at codon 167 and the other surrounding the SNPs at codons 198 and 200. The accuracy of a primer set previously described by Diawara et al. [10] was compared with another set newly designed by our group, for both *T. trichiura* and *N. americanus*. Primers are shown in Table 1 (one of the primers for this PCR was labelled with biotin at the 5′ end). This PCR amplification was carried out using DNA AmpliTools HotSplit Master Mix (BioTools) in a final volume of 50 µl and the primer concentration was 0.5 µM each. PCR conditions were the same for all amplifications although the annealing temperature was different for each species. PCR reactions were performed with the following cycling parameters: an initial incubation at 95 °C for 8 min, followed by 40 cycles at 95 °C for 30 s, an annealing temperature of 59 °C for *T. trichiura* or 62 °C for *N. americanus* for 30 s, 72 °C for 20 s, and a final extension at 72 °C for 5 min. PCR products were monitored by 2% agarose gel electrophoresis. Negative controls (water instead of DNA template) and positive controls (plasmid mixtures) were included in each PCR run. PCR products and sequencing primers were sent to Instituto Aragonés de Ciencias de la Salud (IACS) for pyrosequencing.

**Table 1:** Primers designed by Diawara et al. (2013), and the set designed and optimized by our group (bold italics)

STH	Sense primer (5′-3′)	Antisense primer (5′-3′)	Pyrosequencing primer
Codon 167			
*T. trichiura*	GAGTATCCTGACCGAATTATGACA	(Biot)ACGACGTGAACAGTATCAAACAAC	TGACCGAATTATGACAACT
*N. americanus*	GTGACTGTCTCCAGGTAATTCG	(Biot)CTATAACGTACCTTTGGCGAGGG	GATAGAATCATGTCCTCGT
Codon 198–200			
*T. trichiura*	CGCCTTTTTAGGTTTCAGATACA ***(Biot)GCCTTTTTAGGTTTCAGATACA***	(Biot)GTCTCCGTAAGTTGGTGTTGTTAA ***GTCTCCGTAAGTTGGTGTTGTTAA***	GGTAGAGAACACGGACG ***CGCTTCATTATCTATGCAG***
*N. americanus*	TTTCCGACACTGTGGTTGAG	(Biot)GAGTTCGTTACTAGCCAGCTCACC	GAGAATACAGATGAGACCT ***AGCTTCATTATCAATACAG***

The pyrosequencing methodology at IACS was as follows: up to 40 µl of biotinylated amplicon was incubated with 40 µl of binding buffer (QIAGEN) and 3 µl of streptavidin-coated Sepharose beads (Streptavidin Sepharose^®^ High Performance, GE Healthcare) in a Thermomixer (1400 rpm at RT; Eppendorf). Beads were captured using a Vacuum Prep tool (QIAGEN) and liquids were sequentially passed for 5 s while applying a vacuum to denature the DNA and neutralize the beads as follows: first 70% (v/v) ethanol, followed by 0.2 M NaOH and 10 mM Tris–acetate, pH 7.6. Beads were transferred to a pyrosequencing reaction plate containing 16 pmol sequencing primer in 40 µl of annealing buffer (QIAGEN) and the plate was incubated for 2 min at 80 °C to remove secondary structures. Samples were left at room temperature for 5–10 min. Enzyme mix and substrate were prepared as instructed by the manufacturer (QIAGEN) and, along with dNTPs, added to the reagent cartridge as instructed by the manufacturer (QIAGEN). Pyrosequencing reactions were performed in a PSQ 96MA system (QIAGEN).

#### Specificity of the pyrosequencing assays

The specificity of the pyrosequencing assays was tested using the PCR primers for each STH against several DNA samples obtained from other parasites used as controls, including *T. trichiura*, *N. americanus*, *A. lumbricoides*, *Strongyloides stercoralis*, *Fasciola hepatica*, *F. gigantica*, *Trichostrongylus* spp., *Teladorsagia circumcincta* and *Haemonchus contortus*. We followed the same PCR conditions as for the pyrosequencing PCR described above, and the results were also visualized using 2% agarose gel electrophoresis.

#### Determination of allele frequencies in *T. trichiura* and *N. americanus* pooled eggs

The frequency of the resistance-associated SNPs was estimated in 15 *T. trichiura*- and 15 *N. americanus*-positive samples, all processed by the most efficient protocol after optimizing sample processing. DNA was extracted from the samples using a PowerFecal DNA extraction kit (QIAGEN) following the manufacturer’s instructions. Pyrosequencing was carried out following the protocol described above and samples were analysed in duplicate. In addition, species-specific plasmid mixtures (0%, 50%, and 100% RT/ST) were used as PCR positive controls to later monetize the pyrosequencing assays for both *T. trichiura* and *N. americanus*.

### Statistical analysis

Ct values obtained by real-time polymerase chain reaction (qPCR), and the percentage of the resistance allele frequencies, were expressed as mean values per protocol or per sample, respectively. We compared the mean Ct values to evaluate the differences in stool sample processing between two different protocols. Allele frequencies lower than 10% by pyrosequencing were considered as either absent or below the level of detection of the pyrosequencing. Samples were considered negative by qPCR if they had a Ct value of 51. Initially, the Kolmogorov–Smirnov test was carried out to determine whether data followed a normal distribution. Then, Ct values were compared using the non-parametric Mann–Whitney–Wilcoxon test, and the *p*-value was adjusted using the Bonferroni method. *P*-values < 0.05 were considered significant. All analyses were performed using R software.

## Results

### Optimization of sample processing by measuring *T. trichiura* burden

**Fig. 2:**
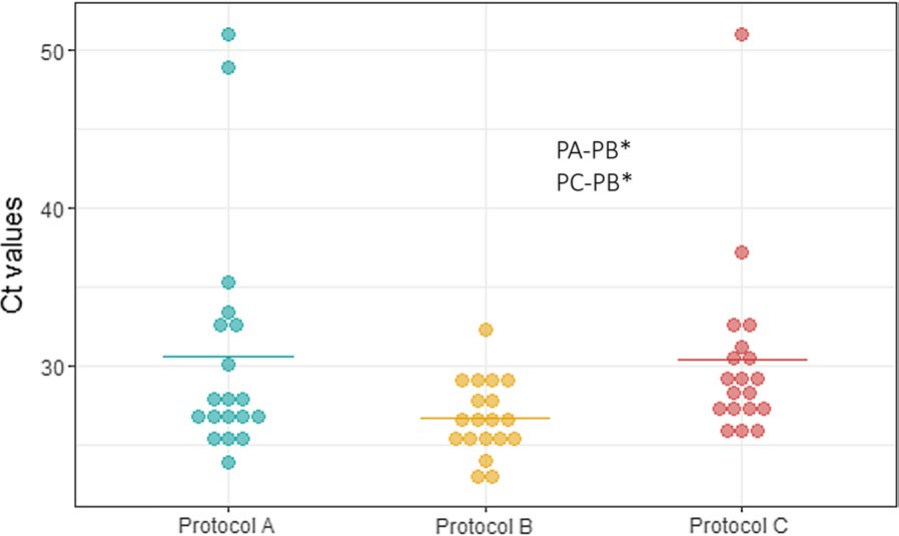
Comparison of Ct values obtained by real-time PCR for each protocol for sample processing. The dot graph shows the Ct values for each protocol. *Differences between protocols were statistically significant (*p* < 0.05). Protocol A (unprocessed stool); Protocol B (egg concentration); and Protocol C (egg concentration and flotation with saturated salt solution)

The amount of *T. trichiura* DNA in the 19 positive samples processed according to the three protocols (57 aliquots) was estimated by qPCR. DNA was amplified in all 19 samples using PB, whereas one sample out of 19 was not amplified by PA and a different sample was not amplified by PC either. A detailed report of the Ct values is shown in Additional file 2: Table S3. Comparing Ct values between aliquots of each sample, the lowest Ct values, or the highest DNA amounts, were found for PB (16/19), followed by PA (3/19). None of the aliquots processed by PC provided the highest DNA concentration when compared to the other two aliquots of the same sample. Ct values are visualized in Fig. 2.

When the mean Ct value was calculated for each protocol, PB showed the lowest value (26.7), followed by PC (30.4) and PA (30.6). Mean Ct values obtained by PB were observed to be significantly lower than those obtained by PA (*p* = 0.002) and PC (*p* < 0.001), whereas no differences were found between PA and PC. This confirms the highest DNA concentration in the aliquots processed by PB.

### Sequencing results for β-tubulin from Mozambican specimens and plasmid constructs

For *T. trichiura*, a final consensus sequence of 530 bp was recovered. Homology with the reference sequence of the β-tubulin of *T. trichiura* isolated in Jamaica (AF034219.1) [24] was 529/530 (99.8%). In *N. americanus*, the size of the consensus sequence was 1259 pb, including the three SNPs; the homology with a sequence resulting from eggs isolated in Tanzania (EF392851.1) [25] was 1252/1259 (99.4%). In both cases, all the nucleotide variations were located in the intron, so no amino acid changes were produced. None of the resistance-associated SNPs were observed after sequencing both species. Based on this information, we designed and produced ST and RT plasmid constructs. Plasmid sequences were confirmed by conventional Sanger sequencing after cloning.

### Standardization of the pyrosequencing assays with plasmid constructs

We first attempted to use the primers described by Diawara et al. [10]. However, we found several inconsistencies when those pyrosequencing primers were used for detecting the SNPs at codons 198 and 200. The pyrosequencing primer showed some failed estimations of the SNP frequencies during the sequencing step (data not shown), so we designed new pyrosequencing primers for both *T. trichiura* and *N. americanus*.

Table 2 is a summary of the pyrosequencing standardization for *T. trichiura* and *N. americanus* species using our new primers. The results represent the arithmetic mean of three repetitions in each experiment, for both *T. trichiura* and *N. americanus*. As observed in Table 2, the frequencies per STH were not different when compared with the SNPs at codons 198 and 200. We decided to validate the primers designed by our group, considering that a pyrosequencing assay naturally accepts a technical background of 10% [26] and slight variations in DNA proportions due to manipulation of the sample when mixtures are prepared. Values for each run are available in Additional file 2: Table S4.

**Table 2:** Optimization of pyrosequencing assays using our own primers and the plasmid constructs previously described

% RT/ST^a^ type according to mixes	% of RT/ST observed by pyrosequencing (mean ± SD)			
	*Tr. trichiura*		*N. americanus*	
	Codon198	Codon 200	Codon 198	Codon 200
0	0 (± 0)	0 (± 0)	0 (± 0)	8.8 (± 3.7)
30	22.2 (± 1.9)	21 (± 3.9)	40.3 (± 6.6)	38 (± 8.3)
50	40.7 (± 2)	38.7 (± 1.2)	53.9 (± 2.6)	52.2 (± 1.1)
70	66.1 (± 2.2)	64.5 (± 1.8)	66.9 (± 4.1)	67.5 (± 0.6)
100	97.1 (± 2.2)	97 (± 2.4)	100 (± 0)	92.8 (± 2.5)

^a^RT/ST: resistant type (RT) and susceptible type (ST) plasmids constructs proportion

### Pyrosequencing specificity

**Fig. 3:**
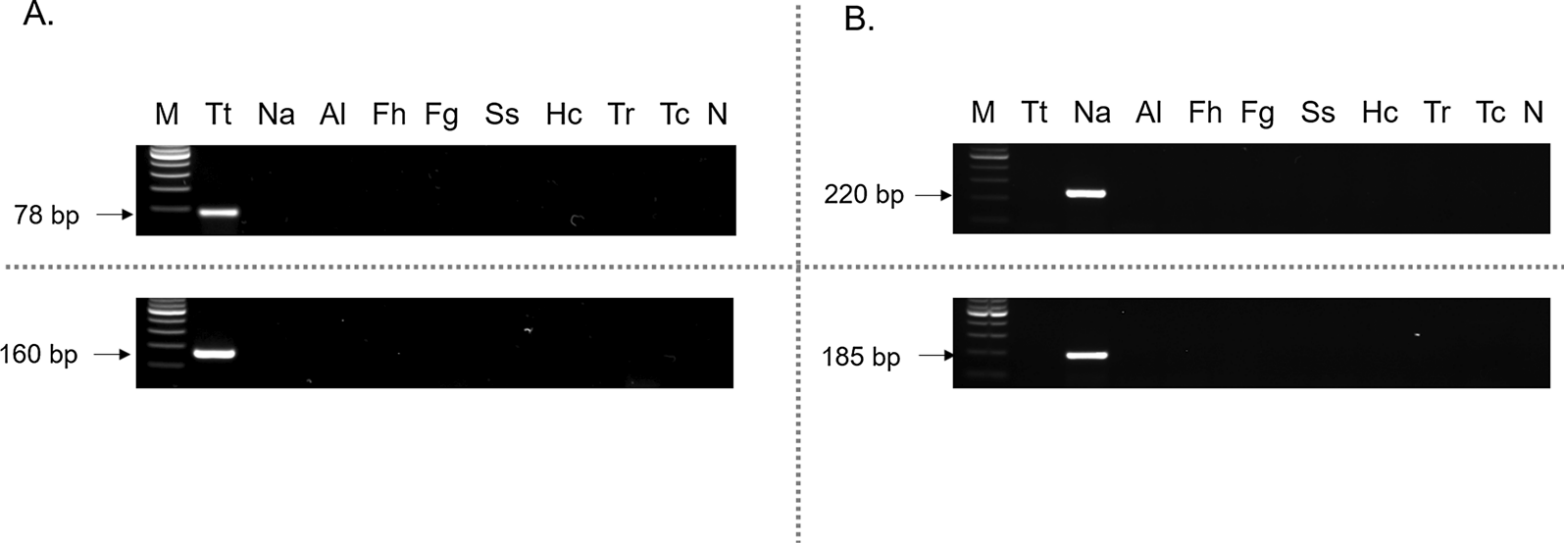
Specificity of the pyrosequencing assays for *T. trichiura* and *N. americanus*. Specificity assessment performed with the primers for **A** *T. trichiura* and **B** *N. americanus* is shown. Upper figures show the PCR reaction using the primers for detecting the SNP at codon 167; down figures refer to the primers for the SNPs at codons 198 and 200. The amplicon size is shown in the right position of the gel and it is expressed in base pair (bp). Lane M, 100 bp DNA ladder; lanes Tt, Na, Al, Fh, Fg, Ss, Hc, Tr, Tc, *T. trichiura*, *N. americanus*, *A. lumbricoides*, *Fasciola hepatica*, *Fasciola gigantica*, *Strongyloides stercoralis*, *Haemonchus contortus*, *Trichostrongylus* spp. and *Teladorsagia circumcincta* DNA samples, respectively; lane N, negative control (no DNA template)

To determine the specificity of the pyrosequencing assays, genomic DNA samples from other helminth parasites were tested for amplification. A positive result was obtained only when using DNA from the specific species corresponding to the pyrosequencing assay, whereas DNA samples from other specimens were not amplified, demonstrating its high specificity (Fig. 3).

### Allele frequencies in *T. trichiura* and *N. americanus* pooled eggs

The resistance SNP frequencies were assessed in the 15 *T. trichiura*- and 15 *N. americanus*-positive samples by pyrosequencing after being processed by PB. Hence, pyrosequencing was carried out in pooled eggs previously concentrated using metallic sieves. Table 3 summarizes the results of the pyrosequencing assays. All frequencies provided for the 15 *T. trichiura*-positive samples for the resistant genotype were below 10%, meaning that resistance-associated SNPs were either absent or below the level of detection of the pyrosequencing assay. For *N. americanus*, one of the 15 samples showed an SNP frequency at codon 198 of 17%, whereas for the remaining samples and SNPs at codons 167 and 200, frequencies were below 10%. In all runs, the control plasmids provided the expected SNP frequencies as previously assessed, thus confirming the correct performance of the experiments and the reliability of the results.

**Table 3:** Pyrosequencing results of 15 *T. trichiura* and 15 *N. americanus* pooled egg samples

*T. trichiura*				*N. americanus*			
Sample ID	Codon 167 (%)	Codon 198 (%)	Codon 200 (%)	Sample ID	Codon 167 (%)	Codon 198 (%)	Codon 200 (%)
T1	1.25	2	0.45	N1	0	0	0
T2	0	2.05	0	N2	0	0	1.15
T3	5.35	2.3	0.55	N3	0	0	0
T4	2.1	2.35	0.65	N4	0	0	1.5
T5	2.75	1.9	0.4	N5	0	0	0
T6	1.3	1.85	0	N6	0	0	1.05
T7	0.95	2.15	0.35	N7	0	1.2	8.3
T8	1.25	1.95	0.4	N8	0	2.05	7.45
T9	0	2.1	0	N9	0	0	0
T10	1.95	2.5	1.65	N10	2.1	17	0
T11	2.6	2.1	0	N11	0	0	6.15
T12	2.3	2.15	0.05	N12	0	0	3.85
T13	1.9	2.75	2.35	N13	0	0	3.85
T14	2.15	5.9	0	N14	0	1.05	3.05
T15	2.35	6.85	0	N15	6.85	0	1.25

Results are expressed as frequencies in percentage of resistant genotype

## Discussion

The low efficacy of benzimidazole treatments is currently considered a serious threat to STH control programmes [5]. Of particular concern is the decreasing efficacy against *T. trichiura* in some countries; globally, egg reduction rates are estimated to have fallen from 72.6% in 1995 to 43.4% in 2015 [5], and a reduction in benzimidazole efficacy against hookworms has also been reported, from 95.6% in 1995 to 77.1% in 2015 [5]. Low anthelmintic efficacy can be due to reasons such as a drug’s pharmacokinetic profile, or improvements in the sensitivity of diagnostic tools after treatment. However, a pronounced decrease in treatment effectiveness after several years of MDA campaigns suggests the genetic selection of resistant strains. Hence, monitoring the emergence of anthelmintic resistance has become of the utmost importance for STH infection control [5, 8].

In veterinary gastrointestinal nematodes, the phenomenon of anthelmintic resistance has been widely studied [27]. In this field, pyrosequencing has been extremely useful for assessing the variation of resistance allele frequencies in isotype-1 β-tubulin before and after benzimidazole administration, thus confirming the implication of these genetic markers in treatment response [17]. The SNP F200Y has been shown to be strongly associated with low efficacy of benzimidazoles, whereas the SNPs F167Y and E198A have been occasionally related with anthelmintic resistance [28]. Besides that, the presence of the E198L polymorphism on its own, a substitution of glutamate to leucine, was very recently related to benzimidazole resistance in *H. contortus* and *T. circumcincta*, two gastrointestinal nematodes infecting ruminants [19, 29]. In humans, only SNP F200Y has been associated with low efficacy of benzimidazoles in *T. trichiura* in one study [10], since the authors observed an increment in the resistant genotype in individual eggs collected after treatment. In *N. americanus*, the resistance markers were detected at codons 167, 198, and 200, although they were not linked to treatment response [10–12, 14]. However, these findings must be interpreted cautiously, since the number of samples analysed was low [9, 10]. Consequently, larger screenings designed to associate the presence of the putative benzimidazole resistance SNPs with drug efficacy are needed.

Previous studies developed different pyrosequencing assays in individual STH eggs. However, the authors reported a high rate of amplification failure of the β-tubulin gene during pyrosequencing, even when applying two rounds of PCR per sample [9, 10]. Generally, DNA detection-based tools for diagnosis incorporate primers targeting highly repeated sequences in the genome [30–32]. Thus, achieving greater sensitivity would be possible if the target sequence is present at a high copy number. By contrast, pyrosequencing must target the β-tubulin gene, which is less repeated than mitochondrial or ribosomal genetic sequences [6], resulting in lower sensitivity than diagnostic-designed PCR. Therefore, improving sample processing and the DNA extraction step is crucial for the highest output of pyrosequencing methods, particularly in low-intensity infections. Our study was focused on the optimization of stool sample processing to increment the amount of parasite DNA in the sample. As seen in several studies, the reason for low PCR efficiency seems to be the robustness of *T. trichiura* eggs, hampering optimal DNA isolation [22, 33]. Thus, achieving the most efficient DNA extraction in *T. trichiura* ensures the overall yield of the protocol for other STH species as well. In order to overcome this drawback, we employed a commercial DNA extraction kit which incorporates an initial mechanical egg disruption step using a bead-beater system, which is crucial for successful DNA isolation from STH eggs [34]. We would also like to highlight the importance of increasing the efficiency of sample processing in anthelmintic efficacy trials. Commonly, anthelmintic treatment reduces helminth burden and, as a consequence, the number of eggs per gram of stool also decreases [35]. Therefore, a step to concentrate the eggs from stool samples before DNA extraction can improve the accuracy of later molecular analysis, particularly in samples collected after treatment. Nevertheless, further work should evaluate different methods for DNA extraction in samples obtained after egg concentration, particularly under field conditions in endemic areas where resources are limited.

Regarding the comparison between protocols for sample processing, egg concentration using only metallic sieves with decreasing pore size (PB) provided the best result. This methodology has also been used for the isolation of gastrointestinal nematode eggs from different animal species, mainly ruminants, for further in vitro studies [36, 37]. Using this method, particles 20–80 µm in size were isolated for later DNA extraction. This size range enables the elimination of all the impurities out of range and the concentration of eggs of all STH, including hookworm and *A. lumbricoides* [38]. This protocol is fast, simple and cheap, and the results for DNA load by real-time PCR demonstrated that it increases the final amount of DNA in the sample. Assigning a Ct value based on the total number of cycles, or the total number of cycles plus 1 (51 in our assay) to negative samples for plotting or statistical analysis has been frequently done in other studies [39]. Moreover, it is interesting to note that the deviation of Ct values provided by real-time PCR was reduced, particularly when compared with unprocessed samples (PA). Therefore, egg concentration also increases the homogeneity of the sample. This may help to reduce the variability between samples and to standardize the protocol to be comparable within studies. However, we would like to highlight that the difference in Ct values between PA and PB in those samples showing amplification (which means excluding negative samples) would be lower (see Fig. 2). Therefore, PA may provide an alternative to PB in programmatic settings due to its simplicity, when the lab capacity is restricted or the number of samples to be processed is large. Lastly, PB could be used not only for later detecting the putative benzimidazole resistance SNPs, but also to support different genotypic studies.

The presence of resistance markers was tested by pyrosequencing, a molecular assay able to measure the frequency of different alleles at specific codons. First, we selected the primers previously described by Diawara and colleagues in 2013 [10]. In that work, the three resistance-associated SNPs were successfully assessed in individual eggs of *T. trichiura*, *N. americanus* and *A. lumbricoides* species. By contrast, in the current study we did not evaluate a specific pyrosequencing method for *A. lumbricoides*, since benzimidazole still seems to be highly efficient against this parasite [4, 5]. However, future work must also move forward on the standardization of assessing benzimidazole resistance in *A. lumbricoides*, because of the risk of the emergence of drug resistance.

When sequencing a fragment of the β-tubulin gene in Mozambican specimens, only SNPs in introns were found when compared to the reference sequences in GenBank. These reference sequences were used in previous studies and correspond to the isotype-1 β-tubulin gene from *N. americanus*, and the β-tubulin gene of *T. trichiura* since this species has only one β-tubulin gene identified in its genome. Actually, *T. trichiura* β-tubulin, although postulated to be involved in benzimidazole resistance, differs substantially from the isotype-1 β-tubulin in other nematodes [40]. Nevertheless, our sequencing results confirm the high degree of conservation of these genes and suggest the potential extensive application of the pyrosequencing primers.

Based on the sequencing results, we designed and produced ST and RT plasmids in order to prepare plasmid DNA mixtures at different proportions. These mixtures simulated different resistance levels potentially present in pooled eggs. Besides that, in our study the plasmid mixtures were tested at the same time as the other samples with two purposes: they were used as positive controls in the first PCR reaction, and they were also used for monitoring the sequencing step, which has been commonly neglected in other studies [9, 10, 16]. Thus, we ensured the correct performance of our experiments and the reliability of our results. At this point, it is important to mention that pyrosequencing equipment reports each result associated with a different level of quality. Diawara et al.’s pyrosequencing primers used in the sequencing step showed some failed-quality analysis and inaccuracy when estimating the frequencies of the SNPs at codons 198 and 200 using plasmids, for both *T. trichiura* and *N. americanus*. Conversely, the sequencing primers designed by our own group were highly precise. We would also like to mention the limitation of the pyrosequencing when trying to assess very low SNP frequencies, since pyrosequencing results below 10% cannot be considered as a low frequency. As an example, we obtained an 8.8% of SNP frequency for *N. americanus* at codon 200 with the susceptible type (ST) plasmid construct. Considering that plasmids were well constructed, the obtained frequency is due to the pyrosequencing technical error.

Thereafter, we used our sequencing primers in all the experiments, although we would note that all outer primers for the first PCR and the sequencing primers for the SNP 167 were the same as previously described by Diawara et al. [10]. In addition, the specificity of the outer primers of the different pyrosequencing assays was evaluated for the first time. No cross-amplification was found when using genomic DNA extracted from several helminth parasites as controls. Assessing the specificity against other pathogens is necessary if pooled eggs are evaluated since co-infections are usual in endemic areas [26]. Non-specific amplification may provide inaccurate SNP frequencies. Thus, primer specificity was confirmed, which allows the use of the same processing method and subsequently the same DNA for pyrosequencing any sample, even in the case of co-infections.

Once the pyrosequencing assays were evaluated, we estimated the frequency of the resistance-associated SNPs from pooled egg samples. The SNP frequencies were successfully evaluated although we only found the resistant genotype in one *N. americanus*-positive sample at codon 198. Stool samples were collected across the Manhiça district, which consists of several communities and more than 200,000 inhabitants. Therefore, the proportion of the stool samples that we have analysed is quite low when compared to the total population in the study area, and we cannot infer the real prevalence of this SNP across the different communities. Besides that, the presence of putative resistance SNPs has been also reported at very low frequencies in previous studies [12]. Future work must assess the real prevalence of the three putative benzimidazole resistance SNPs in the Manhiça district and clarify the real implications of these variations in the response to benzimidazole treatment.

Recently, the implication of these SNPs in the development of benzimidazole resistance in STH has been questioned, since some studies reported resistant strains of *T. trichiura* and hookworm without these markers [41, 42]. For this reason, genome-wide approaches have been proposed as the most suitable methods for clarifying the molecular mechanism of anthelmintic resistance in STH and defining new genetic markers [43]. Regardless of the resistance marker, pyrosequencing may be later used for evaluating its frequency in different parasite subpopulations, since this method is more cost-effective and faster than genome-wide approaches, once the resistance marker is well defined.

Lastly, we would like to highlight the importance of concentrating the eggs in the stool sample when assessing resistance markers. Each single egg could show a resistant or susceptible homozygote genotype, or a heterozygote genotype. The initial egg concentration of the sample may provide the most accurate SNP frequency of the parasite subpopulation within one infected patient. The higher the number of individuals screened, the more representative the data of the parasite populations. In this study, we show optimization of the protocol for sample processing and the standardization of pyrosequencing, which could be implemented in large-scale anthelmintic response monitoring or drug efficacy trials. Further work must comprehensively evaluate these assays under field conditions in order to completely validate these pyrosequencing methods.

## Supplementary Information

**Additional file 1.** Standard operation procedure of the three protocols for sample processing.

**Additional file 2.** Additional Tables S1, S2, S3 and S4.

## Data Availability

All data generated or analysed during this study are included in this published article, and its additional files.

## References

[1] World Health Organization. https://www.who.int/neglected_diseases/resources/who_wer9350/en/. Accessed 20 Dec 2020.

[2] World Health Organization. Guideline: preventive chemotherapy to control soil-transmitted helminth infections in at-risk population groups. Geneva 2017. http://www.who.int/iris/handle/10665/258983. Accessed 20 Dec 2020. 29578660

[3] Keiser J, Utzinger J (2008). Efficacy of current drugs against soil-transmitted helminth infections: systematic review and meta-analysis. JAMA.

[4] Moser W, Schindler C, Keiser J (2017). Efficacy of recommended drugs against soil transmitted helminths: systematic review and network meta-analysis. BMJ.

[5] Schulz JD, Moser W, Hürlimann E, Keiser J (2018). Preventive Chemotherapy in the fight against soil-transmitted helminthiasis: achievements and limitations. Trends Parasitol.

[6] Kotze AC, Hunt PW, Skuce P, von Samson-Himmelstjerna G, Martin RJ, Sager H (2014). Recent advances in candidate-gene and whole-genome approaches to the discovery of anthelmintic resistance markers and the description of drug/receptor interactions. Int J Parasitol Drugs Drug Resist.

[7] Gandasegui J, Martínez-Valladares M, Grau-Pujol B, Krolewiecki AJ, Balaña-Fouce R, Gelaye W (2020). Role of DNA-detection–based tools for monitoring the soil-transmitted helminth treatment response in drug-efficacy trials. PLoS Negl Trop Dis.

[8] Vlaminck J, Cools P, Albonico M, Ame S, Ayana M, Bethony J (2018). Comprehensive evaluation of stool-based diagnostic methods and benzimidazole resistance markers to assess drug efficacy and detect the emergence of anthelmintic resistance: a starworms study protocol. PLoS Negl Trop Dis.

[9] Diawara A, Drake LJ, Suswillo RR, Kihara J, Bundy D, Scott ME (2009). Assays to detect beta-tubulin codon 200 polymorphism in *Trichuris trichiura* and *Ascaris lumbricoides*. PLoS Negl Trop Dis.

[10] Diawara A, Halpenny CM, Churcher TS, Mwandawiro C, Kihara J, Kaplan RM (2013). Association between response to albendazole treatment and β-tubulin genotype frequencies in soil-transmitted helminths. PLoS Negl Trop Dis.

[11] Diawara A, Schwenkenbecher JM, Kaplan RM, Prichard RK (2013). Molecular and biological diagnostic tests for monitoring benzimidazole resistance in human soil-transmitted helminths. Am J Trop Med Hyg.

[12] Zuccherato LW, Furtado LF, Medeiros CDS, Pinheiro CDS, Rabelo ÉM (2018). PCR-RFLP screening of polymorphisms associated with benzimidazole resistance in *Necator americanus* and *Ascaris lumbricoides* from different geographical regions in Brazil. PLoS Negl Trop Dis.

[13] Schwenkenbecher JM, Albonico M, Bickle Q, Kaplan RM (2007). Characterization of beta-tubulin genes in hookworms and investigation of resistance-associated mutations using real-time PCR. Mol Biochem Parasitol.

[14] Orr AR, Quagraine JE, Suwondo P, George S, Harrison LM, Dornas FP (2019). Genetic markers of benzimidazole resistance among human hookworms (*Necator americanus*) in Kintampo North Municipality. Ghana Am J Trop Med Hyg.

[15] Rashwan N, Bourguinat C, Keller K, Gunawardena NK, de Silva N, Prichard R (2016). Isothermal diagnostic assays for monitoring single nucleotide polymorphisms in *Necator americanus* associated with benzimidazole drug resistance. PLoS Negl Trop Dis.

[16] Esteban-Ballesteros M, Rojo-Vázquez FA, Skuce PJ, Melville L, González-Lanza C, Martínez-Valladares M (2017). Quantification of resistant alleles in the β-tubulin gene of field strains of gastrointestinal nematodes and their relation with the faecal egg count reduction test. BMC Vet Res.

[17] Barrère V, Alvarez L, Suarez G, Ceballos L, Moreno L, Lanusse C (2012). Relationship between increased albendazole systemic exposure and changes in single nucleotide polymorphisms on the β-tubulin isotype 1 encoding gene in *Haemonchus contortus*. Vet Parasitol.

[18] Hinney B, Schoiswohl J, Melville L, Ameen VJ, Wille-Piazzai W, Bauer K (2020). High frequency of benzimidazole resistance alleles in trichostrongyloids from Austrian sheep flocks in an alpine transhumance management system. BMC Vet Res.

[19] Mohammedsalih KM, Krücken J, Khalafalla A, Bashar A, Juma FR, Abakar A (2020). New codon 198 β-tubulin polymorphisms in highly benzimidazole resistant *Haemonchus contortus* from goats in three different states in Sudan. Parasit Vectors.

[20] Katz N, Chaves A, Pellegrino J (1972). A simple device for quantitative stool thick-smear technique in *Schistosomiasis mansoni*. Rev Inst Med Trop Sao Paulo.

[21] Phuphisut O, Yoonuan T, Sanguankiat S, Chaisiri K, Maipanich W, Pubampen S (2014). Triplex polymerase chain reaction assay for detection of major soil-transmitted helminths, *Ascaris lumbricoides, Trichuris trichiura, Necator americanus*, in fecal samples. Southeast Asian J Trop Med Public Health.

[22] Kaisar MMM, Brienen EAT, Djuardi Y, Sartono E, Yazdanbakhsh M, Verweij JJ (2017). Improved diagnosis of *Trichuris trichiura* by using a bead-beating procedure on ethanol preserved stool samples prior to DNA isolation and the performance of multiplex real-time PCR for intestinal parasites. Parasitology.

[23] Untergasser A, Nijveen H, Rao X, Bisseling T, Geurts R, Leunissen JAM (2007). Primer3Plus, an enhanced web interface to Primer3. Nucleic Acids Res.

[24] Bennett AB, Barker GC, Bundy DA (1999). A beta-tubulin gene from *Trichuris trichiura*. Mol Biochem Parasitol.

[25] Albonico M, Wright V, Bickle Q (2004). Molecular analysis of the beta-tubulin gene of human hookworms as a basis for possible benzimidazole resistance on Pemba Island. Mol Biochem Parasitol.

[26] Ramünke S, Melville L, Rinaldi L (2016). Benzimidazole resistance survey for Haemonchus, Teladorsagia and Trichostrongylus in three European countries using pyrosequencing including the development of new assays for Trichostrongylus. Int J Parasitol Drugs Drug Resist.

[27] Jourdan PM, Lamberton PHL, Fenwick A, Addiss DG (2018). Soil-transmitted helminth infections. Lancet.

[28] Jimenez Castro PD, Howell SB, Schaefer JJ, Avramenko RW, Gilleard JS, Kaplan RM (2019). Multiple drug resistance in the canine hookworm *Ancylostoma caninum*: an emerging threat?. Parasit Vectors.

[29] Martínez-Valladares M, Valderas-García E, Gandasegui J, Skuce P, Morrison A, Castilla Gómez de Agüero V (2020). *Teladorsagia circumcincta* beta tubulin: the presence of the E198L polymorphism on its own is associated with benzimidazole resistance. Parasit Vectors.

[30] Fernández-Soto P, Gandasegui J, Sánchez Hernández A, López Abán J, Vicente Santiago B, Muro A (2014). A loop-mediated isothermal amplification (LAMP) assay for early detection of *Schistosoma mansoni* in stool samples: a diagnostic approach in a murine model. PLoS Negl Trop Dis.

[31] Polley SD, Mori Y, Watson J, Perkins MD, González IJ (2010). Mitochondrial DNA targets increase sensitivity of malaria detection using loop-mediated isothermal amplification. J Clin Microbiol.

[32] Gandasegui J, Fernández-Soto P, Carranza-Rodríguez C, Pérez-Arellano JL, Vicente B, Muro A (2015). The Rapid-Heat LAMPellet Method: a potential diagnostic method for human urogenital schistosomiasis. PLoS Negl Trop Dis.

[33] Mejia R, Vicuña Y, Broncano N, Sandoval C, Vaca M (2013). A novel, multi-parallel, real-time polymerase chain reaction approach for eight gastrointestinal parasites provides improved diagnostic capabilities to resource-limited at-risk populations. Am J Trop Med Hyg.

[34] Ayana M, Cools P, Mekonnen Z, Biruksew A, Dana D, Rashwan N (2019). Comparison of four DNA extraction and three preservation protocols for the molecular detection and quantification of soil-transmitted helminths in stool. PLoS Negl Trop Dis.

[35] Vlaminck J, Cools P, Albonico M, Ame S, Ayana M, Cringoli G (2019). Therapeutic efficacy of albendazole against soil-transmitted helminthiasis in children measured by five diagnostic methods. PLoS Negl Trop Dis.

[36] Esteban-Ballesteros M, Sanchis J, Gutiérrez-Corbo C, Balaña-Fouce R, Rojo-Vázquez FA (2019). In vitro anthelmintic activity and safety of different plant species against the ovine gastrointestinal nematode *Teladorsagia circumcincta*. Res Vet Sci.

[37] Giovanelli F, Mattellini M, Fichi G, Flamini G, Perrucci S (2018). In vitro anthelmintic activity of four plant-derived compounds against sheep gastrointestinal nematodes. Vet Sci.

[38] World Health Organization. Bench aids for the diagnosis of intestinal parasites. 1994. World Health Organization. https://apps.who.int/iris/handle/10665/37323. Accessed 20 Dec 2020.

[39] Rachow A, Zumla A, Heinrich N, Rojas-Ponce G, Mtafya B, Reither K (2011). Rapid and accurate detection of Mycobacterium tuberculosis in sputum samples by Cepheid Xpert MTB/RIF assay—a clinical validation study. PLoS ONE.

[40] Demeler J, Krüger N, Krücken J, von der Heyden VC, Ramünke S, Küttler U (2013). Phylogenetic characterization of β-tubulins and development of pyrosequencing assays for benzimidazole resistance in cattle nematodes. PLoS ONE.

[41] Furtado LFV, de Aguiar PHN, Zuccherato LW, Teixeira TTG, Alves WP, da Silva VJ (2019). Albendazole resistance induced in *Ancylostoma ceylanicum* is not due to single-nucleotide polymorphisms (SNPs) at codons 167, 198, or 200 of the beta-tubulin gene, indicating another resistance mechanism. Parasitol Res.

[42] Matamoros G, Rueda MM, Rodríguez C, Gabrie JA, Canales M, Fontecha G (2019). High endemicity of soil-transmitted helminths in a population frequently exposed to albendazole but no evidence of antiparasitic resistance. Trop Med Infect Dis.

[43] Doyle SR, Cotton JA (2019). Genome-wide approaches to investigate anthelmintic resistance. Trends Parasitol.

