# Supplementary material for: Improving stool sample processing and pyrosequencing for quantifying benzimidazole resistance alleles in Trichuris trichiura and Necator americanus pooled eggs
Source: Parasit Vectors. 2021 Sep 25;14:490. doi: 10.1186/s13071-021-04941-w (PMC8466976; doi:10.1186/s13071-021-04941-w)
Supplement: Supplementary file 1 — Additional file 1. Standard operation procedure of the three protocols for sample processing. [file 13071_2021_4941_MOESM1_ESM.docx]

**PROTOCOLS FOR STOOL SAMPLE PROCESSING**

**Material**

Equipment

- Scale
- Vortex
- Centrifuge for 15 mL tubes
- Micropipette or plastic pipettes
- Printer for labels

Consumables

- Disposable gloves (latex or nitrile)
- Wooden spatulas to transfer, weight and mix stool (single use)
- Metallic sieve – pore size 150 µm
- Metallic sieve – pore size 80 µm
- Metallic sieve – pore size 20 µm
- Small plastic funnels (must fit in the 15 mL falcon tubes)
- Rubber tube
- Glass beads 6 mm
- Screw cap Micro tubes 2 mL
- Falcon tubes – 15 mL
- 1000 µl tips (for micropipette)
- Labels suitable for cryopreservation
- Plastic racks for 2 mL tubes
- Plastic racks for 15 mL tubes
- Tap water
- Saturated salt solution
- 1% bleach working solution (to clean sieves and benches)

**Protocol A**

1. Exhaustively homogenize the stool sample using a spatula.
2. Weight 250 mg of the stool in to a 2 mL screw cap tube.
3. Preserve it at -80ª. Proceed with the DNA extraction.

**Protocol B**

1. Weight 3 g of homogenized stool sample and proceed with the egg concentration steps straightaway.
2. Assemble the three metallic sieves in decrescent order of pore size (150-80-20 µm). The sieve with 150 µm pore size must be on the top, followed by the 80 µm sieve and finally the 20 µm sieve. Once assembled, put them in the sink under the tap.


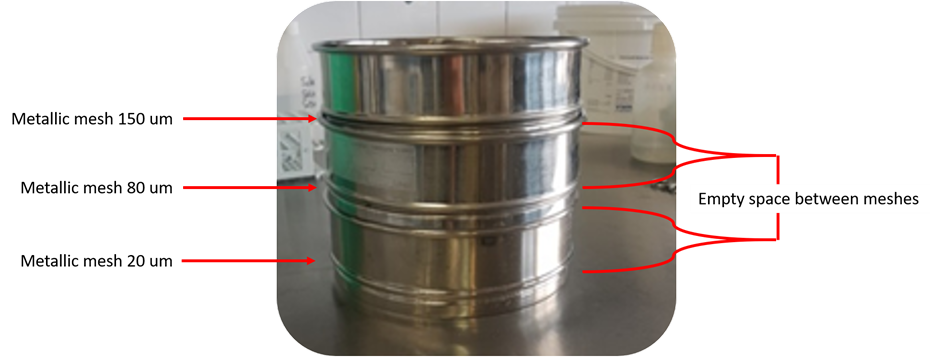


1. Fit the rubber tube in the tap to add high-pressure water.
2. In the same plastic container where samples were stored, add 20-30 glass beads and shake vigorously in order to disaggregate and dissolve the stool in the water.

1. Pour the disaggregated faeces on the top of the three sieves (150 µm) and wash them with tap water. Pressing the rubber tube increases the water pressure and may help to clean the stool. Be aware to not fill the space between sieves, water must completely pass by the sieves and this could take some time depending on the characteristics of the sample. Slightly move the sieves one by one, up and down, to facilitate the sample passing through the sieves. Keep washing until all particles smaller than the pore size have passed through each sieve.
2. The sediment retained in the 20 µm sieve contains the STH eggs and a little amount of impurities.
3. Fit the plastic funnels in the 15 mL falcon tubes and use the wash-bottle previously filled with tap water to rinse the sieve and collect the retained sediment into the falcon tube.
4. Centrifuge the tubes at 2000 rpm for 5 min and discard the supernatant.
5. Transfer all the sediment using a pipette to a 2 mL screw cap tubes.
6. Close the tubes firmly.
7. Before storage, always check that the tube has a label with the correct ID, correlated to the original container with the stool sample.
8. Freeze the samples at -80°C as soon as possible (within 24 hours).
9. Proceed with the DNA extraction protocol.
10. A critical step to avoid cross contamination is mesh washing between samples sieving. Use 1% bleach working solution to clean the meshes. Afterwards, flip the sieves and wash them using high-pressure tap water, and verify that there is not any particle retained in the pores of the mesh. Having two sets of the three sieves would be recommendable. In this way, eggs can be concentrated and in the meantime the other set of sieves could be kept with 1% bleach solution.

**Protocol C**

1. Continue from step 8 of Protocol B.
2. Mixed the sediment with 10 mL of saturated salt solution, mix and centrifuge for 2000 rpm for 5 min.
3. Recover the top 5 mL of the supernatant into a new clean 15 mL falcon tube.
4. Add 10 mL of distilled water and mix.
5. Centrifuge the tubes at 2000 rpm for 5 min and discard the supernatant.
6. Continue with step 9 of Protocol B.
