# Supplementary material for: Improving stool sample processing and pyrosequencing for quantifying benzimidazole resistance alleles in Trichuris trichiura and Necator americanus pooled eggs
Source: Parasit Vectors. 2021 Sep 25;14:490. doi: 10.1186/s13071-021-04941-w (PMC8466976; doi:10.1186/s13071-021-04941-w)
Supplement: Supplementary file 2 — Additional file 2. Additional Tables S1, S2, S3 and S4. [file 13071_2021_4941_MOESM2_ESM.docx]

**ADDITIONAL FILE 1 - SUPPLEMENTARY TABLES**

**Table S1.** Primers used for sequencing the β tubulin fragment which includes the three condones with the resistance associated SNPs.

| **Species** |  | **Sequence** | **Tm ºC** | **Annealing pos.*** | **Product size** |
| --- | --- | --- | --- | --- | --- |
| ***T. trichiura*** | Pair 1 | Fw- AACTGGGAGTGGAATGGGTA  Rev-GAAAGCGTAGGCATGTCGTT | 59.3  60.3 | 887-907  1386-1406 | 348 bp |
|  | Pair 2 | Fw- ATTTCGAGGAACAGCACGTT  Rev- GGTTGCCGAAACCAAATGAT | 59.7  62 | 1070-1090  1417-1437 | 500 bp |
| ***N. americanus* fragment A** | Pair 1 | Fw- TCGCAAAGAAGCTGAAGGAT  Rev- CAGCCAGTACGCACTCGTAA | 60.1  60.1 | 847-867  1607-1627 | 761 bp |
|  | Pair 2 | Fw-GTTCGCAAAGAAGCTGAAGG  Rev- GGACAAGGCGCAGTTAGTTC | 60.1  59.9 | 845-865  1629-1649 | 785 bp |
| ***N. americanus* fragment B** | Pair 3 | Fw-AACCGGCTTCAACTTCCTTT  Rev- ATTCAACTGACCGGGGAAG | 60.1  59.9 | 1430-1450  2127-2146 | 698 bp |
|  | Pair 4 | Fw- CGGCTTCAACTTCCTTTGAC  Rev-GGGGAAGCGAAGACAGGTAG | 59.9  61.1 | 1433-1453  2115-2135 | 683 bp |

***:** The annealing position refers to the Genbank sequences of the b-tubulin for each STH: *T. trichiura* GenBank code AF034219 and *N. americanus* GenBank code EF392851.

**Table S2.** PCR conditions for each primers pair, which were used for sequencing the β tubulin of the STH species from Mozambique.

| **Species and primer pair** |  |  |  |  |  |  |
| --- | --- | --- | --- | --- | --- | --- |
| ***T. trichiura* pair 1** | Temperature | 95 ºC | 95 ºC | 59.8 ºC | 72 ºC | 72 ºC |
|  | Time | 6 min | 30 s | 30 s | 30 s | 8 min |
|  | Cycles | x 1 | x 40 | | | x 1 |
| ***T. trichiura* pair 2** | Temperature | 95 ºC | 95 ºC | 60.8 ºC | 72 ºC | 72 ºC |
|  | Time | 7 min | 40 s | 40 s | 36 s | 8 min |
|  | Cycles | x 1 | x 40 | | | x 1 |
| ***N. americanus* fragment A (pair 1 and 2) and fragment B (pair 1 and 2)** | Temperature | 95 ºC | 95 ºC | 63 ºC | 72 ºC | 72 ºC |
|  | Time | 7 min | 40 s | 40 s | 48 s | 9 min |
|  | Cycles | x 1 | x 40 | | | x 1 |

**Table S3.** Ct values for each sample and for each protocol after the application of the real-time PCR

|  | Protocol A | Protocol B | Protocol C |
| --- | --- | --- | --- |
| Sample 1 | negative | **29.2** | 32.4 |
| Sample 2 | 32.3 | **29.0** | 31.2 |
| Sample 3 | 30.1 | **29.1** | 29.3 |
| Sample 4 | 33.4 | **32.3** | 37.2 |
| Sample 5 | 27.8 | **27.0** | 30.9 |
| Sample 6 | **28.1** | 29.0 | 30.1 |
| Sample 7 | **25.4** | 25.9 | negative |
| Sample 8 | 35.3 | **27.9** | 32.9 |
| Sample 9 | 28.1 | **27.7** | 29.1 |
| Sample 10 | 33.0 | **25.7** | 27.8 |
| Sample 11 | 26.7 | **26.2** | 29.4 |
| Pool 1 | 27.1 | **26.4** | 28.7 |
| Pool 2 | 27.0 | **25.1** | 25.8 |
| Pool 3 | 27.0 | **26.4** | 28.0 |
| Pool 4 | **23.9** | 24.0 | 26.0 |
| Pool 5 | 26.5 | **25.2** | 27.2 |
| Pool 6 | 25.3 | **25.0** | 26.9 |
| Pool 7 | 25.5 | **22.8** | 25.9 |
| Pool 8 | 48.9 | **23.3** | 27.6 |

**Table S4.** Values of each replicate during the optimization of pyrosequencing primers designed by our own group.

| **% RT/ST* type according to mixes** | **% of RT/ST observed by pyrosequencing** | | | | | | | | | | | |
| --- | --- | --- | --- | --- | --- | --- | --- | --- | --- | --- | --- | --- |
|  | ***Trichuris trichiura*** | | | | | | ***Necator americanus*** | | | | | |
|  | **Condon198** | | | **Codon 200** | | | **Codon 198** | | | **Codon 200** | | |
| **0** | 0 | 0 | 0 | 0 | 0 | 0 | 0 | 0 | 0 | 8.6 | 11.5 | 6.2 |
| **30** | 21.0 | 21.3 | 24.4 | 25.3 | 18.7 | 20.0 | 46.8 | 33.6 | 40.6 | 29.1 | 39.7 | 45.4 |
| **50** | 38.7 | 42.7 | 40.8 | 38.7 | 42.7 | 40.8 | 51.4 | 53.7 | 56.6 | 53.4 | 51.4 | 51.8 |
| **70** | 68.5 | 64.3 | 65.6 | 64.8 | 62.5 | 66.1 | 72.3 | 66.9 | 64.2 | 68.2 | 67.3 | 67.1 |
| **100** | 98.9 | 97.8 | 94.6 | 97.8 | 98.9 | 94.3 | 100 | 100 | 100 | 90.3 | 92.9 | 95.2 |

***: RT/ST:** resistant type (RT) and susceptible type (ST) plasmids constructs proportion
